# Study design factors influencing patients’ willingness to participate in clinical research: a randomised vignette-based study

**DOI:** 10.1186/s12874-020-00979-z

**Published:** 2020-04-26

**Authors:** Angèle Gayet-Ageron, Sandrine Rudaz, Thomas Perneger

**Affiliations:** Division of Clinical Epidemiology and Department of health and community medicine, Geneva University Hospitals and Faculty of Medicine, 6 Rue Gabrielle Perret-Gentil, 1211, 14 Geneva, Switzerland

**Keywords:** Patient participation, Clinical research, Controlled trials, Study design, Randomization, Clinical equipoise

## Abstract

**Background:**

High patient participation in clinical research reduces selection bias and ensures the generalizability of study findings. We explored study-related factors that may influence patients’ willingness to participate in research.

**Methods:**

We submitted by mail two vignettes that described clinical research studies – a drug trial and a diagnostic study – to patients recently discharged from hospital and assessed their willingness to participate. We used a factorial design to randomly allocate three study attributes per vignette: in the drug trial, presumed superiority of new drug versus equipoise, public versus industry funding, and random versus non-random treatment allocation; in the diagnostic study, common versus rare disease, genetic versus protein analysis, and automatic reporting of results versus reporting on request.

**Results:**

Of 2600 patients contacted, 1140 (44%) participated. Globally, willingness to participate in a drug trial was lower than in a diagnostic study (44.8% vs. 76.2%; *P* < 0.001). In the drug trial, participation was significantly higher when the new drug was presented as presumably better than the old (vs. equipoise) and when the study was funded by public sources (vs. industry), but was not affected by the allocation method. None of the factors tested in the diagnostic study was associated with participation.

**Conclusions:**

Patients were more likely to participate in a hypothetical observational diagnostic study than in a hypothetical drug trial. Participation in the trial was lower when clinical equipoise was expressed and when the trial was funded by industry. These results suggest that some features of study design can influence participation.

## Background

Clinical studies depend on the participation of potential research subjects. Difficult patient recruitment is the main reason for abandoning a clinical research study [1]. Low participation rates may lead to selection bias and impede the generalizability of study findings drawn from non-representative samples [2]. In addition, a smaller sample size reduces the precision of estimates and statistical power. Understanding the study factors associated with willingness to participate in clinical research is crucial to help guide efforts aimed at improving participation.

Several studies have explored the factors associated with patient participation in clinical trials. For example, sicker patients were more likely to participate in research than healthier patients [3]. Patients expressed altruistic motivations, but also the hope of potential personal benefits from participation in cancer trials for example [4–6], but participation was not associated with tumor stage/type, age or gender [7]. Among factors related to the study design and logistics, prior approval from an ethics committee [8] and public release of results [9] were associated with greater participation, but random allocation of interventions [10, 11], placebo use [12, 13], complex or inadequate study information [14], and personal inconvenience (e.g. extra appointments, burden of intervention) [12] were associated with lower participation.

Most previous studies used an observational design with quantitative or qualitative approaches [3–6, 9–14]. Here, we conducted an experimental study among a representative sample of patients discharged from a Swiss university hospital to determine the study factors that influence patients’ willingness to participate in clinical research. The primary aim of the survey was to evaluate patient opinion about a biobank project at the hospital [15] and we present here an ancillary study.

## Methods

### Study design, setting and participants

We conducted a cross-sectional study between 1 March and 31 March 2014 among a random sample of all patients (*n* = 3520). hospitalised at least 24 h at Geneva University Hospitals, a 2000-bed public teaching hospital in Geneva, Switzerland, with > 48,000 admissions per year, representing > 670,000 hospitalization-days. We only included adults > 18 years old and excluded patients who resided outside Switzerland for practical reasons (pre-stamped envelopes, avoidance of any problems due to possible differences in policies between Switzerland and France or other neighbouring countries).

### Questionnaire and clinical research vignettes

All participants received a survey package by mail. It included an introductory letter presenting the purposes of the research, a 23-item questionnaire, a form that allowed the patient to give the reasons for non-participation (inability to complete the questionnaire, poor health conditions or simple refusal to participate), and a pre-paid return envelope. The first mailing was sent 8–12 weeks after discharge and two reminders were sent during the next 2 months.

The questionnaire had three parts. The first part included seven items assessing the participant’s opinion on various aspects of research. The second part presented four clinical vignettes, including two presented here (Additional file 1). The two other vignettes were related to a biobank project and did not explore specifically factors associated with participation to clinical research [15]. Each vignette tested three binary factors that were randomly attributed using a factorial design, thus 8 versions were created for each vignette. The order of presentation of the vignettes was the same in all eight versions of the survey. The first vignette presented a clinical trial assessing the efficacy of a new drug and the second presented an observational study assessing the performance of a new laboratory test. The third part of the questionnaire collected information on participant characteristics.

### Primary outcomes

At the end of each vignette, respondents were asked whether they would agree to participate (primary outcome) on a 5-point Likert scale: 1) “*I would certainly refuse*”; 2) “*I would probably refuse*”; 3) “*I am not sure*”; 4) “*I would probably participate*”; and 5) “*I would certainly participate*”.

### Experimental factors and study hypotheses

In the clinical trial vignette, we tested the following factors: 1) belief of greater effectiveness of the new drug (absence of equipoise, an ethical principle for clinical trials) vs. clinical equipoise; 2) random allocation of study drugs (important methodologic attribute of trials) vs. medical decision (poor methodologic attribute); and 3) public research funding vs. research financed by a drug company. All versions of this vignette mentioned side-effects of the new drug (digestive symptoms and dizziness). We anticipated that a belief of the greater effectiveness of the study drug, drug allocation by medical decision and a public source of funding would be associated with a greater propensity to participate. In the diagnostic test vignette, we tested the three following factors: 1) rare disease (1–2 among 10,000 inhabitants in Switzerland, not named) vs. frequent disease (heart disease); 2) genetic vs. blood protein analyses; and 3) automatic reporting of test results to the participant vs. reporting only upon request. We anticipated that a frequent disease (more likely to affect the patient personally), protein analysis (rather than a genetic test that may be perceived as more intrusive), and automatic reporting of results (hence a greater personal benefit) would be associated with a greater propensity to participate.

### Other variables collected

These variables included patient age, gender, country of birth, level of education, number of children, and self-rated health status. Finally, the questionnaire included items assessing the patient’s opinion on genetic research (defined by the study of human DNA found in all cells) and on the utility of clinical research, his/her past participation in clinical research, and if he/she was a blood or organ donor (as an indicator of altruism).

### Power calculation

The study was initially designed to assess the precision of the opinion of patients regarding a hospital-based biobank and expected to be 70 ± 2.5%. As the participation rate was expected to be 50%, a sample size of 2600 was calculated [15]. Using the expected sample size, we estimated to be able to detect odds ratios of 0.74 or 1.39 for the willingness to participate in one category relative to the reference category of the experimental factor with a power of 80%.

### Statistical analysis

The two vignettes were analysed separately. For each vignette, we estimated the intent to participate by grouping “*I would certainly participate*” with “*I would probably participate*” and estimated the 95% confidence interval (CI) using the Clopper-Pearson’s exact binomial method. We tested if there was a difference in the willingness to participate between the first and second vignettes using McNemar’s Chi-2 test. We then assessed the two primary outcomes using the original 5-point Likert scale (ordinal format) and we tested the correlation of answers in the two vignettes by a Spearman coefficient (rho). All covariates and responses to vignettes were compared among the eight versions of the survey using Student’s t-test for continuous variables and the Chi-2 test for categorical variables. We used an ordered logistic regression model for each vignette to estimate the association between the likelihood of participation (dependent variable) and the three dichotomous experimental factors (independent variables).

In a second step, we constructed two new multivariable models including the three experimental factors, plus four pre-specified variables that captured the influence of altruistic behavior (previous participation in a clinical study, blood donor and/or potential organ donor, opinion toward clinical research ranging from “very negative” to “very positive”), and patient’s self-rated health status categorised as excellent/very good vs. good/fair/poor in the intention to participate to clinical research. All analyses were performed using Stata version intercooled 15 (Stata Corp., College Station, TX, USA). Statistical significance was defined as *P* < 0.05 (two-sided).

## Results

### Patient characteristics

Among a total of 3520 eligible patients, we randomly selected 2600 patients; 1140 (43.8%) returned the questionnaires. Among these, 1125 (98.7%) responded to at least one vignette and were included in the current analysis. Reasons for non-participation were refusal (*n* = 32), death (*n* = 22) and failure to return the questionnaire (*n* = 1406). The mean age of respondents was 60 years; 618 (56%) respondents were women (Table 1). A comparison of the mean age (59.3 ± 21.0 standard deviation) and the proportion of women (55.6%) with the initial eligible population of hospitalised patients was similar. Most respondents were born in Switzerland; 56% had completed elementary school or an apprenticeship, 54% were married, and 79% had at least one child. A minority considered their health as excellent or very good (23%) and one-third had been hospitalised in the last 6 months. We did not find any difference between the groups of patients randomly allocated to the eight versions of the questionnaire (data not shown).

**Table 1:** Respondents’ characteristics

Variables	Respondents (***N*** = 1125^**†**^)
Female gender, n (%)	618 (55.7)
Mean age (standard deviation, median)	60.0 (±19.4, 63)
Categories of age (yr), n (%)	
< 40	210 (19.8)
40–59	268 (25.2)
60–74	288 (27.1)
≥ 75	296 (27.9)
Country of birth, n (%)	
Switzerland	575 (51.9)
Other European countries	361 (32.6)
Other countries	171 (15.5)
Level of education, n (%)	
Elementary school	269 (24.4)
Apprenticeship	350 (31.7)
Secondary school	119 (10.8)
Professional school	141 (12.8)
University	224 (20.3)
Marital status, n (%)	
Married	594 (53.8)
Single, divorced, separated, widowed	511 (46.2)
Children, n (%)	
Yes	867 (78.6)
No	236 (21.4)
Self-rated health status, n (%)	
Excellent	67 (6.1)
Very good	189 (17.2)
Good	519 (47.1)
Fair	251 (22.8)
Poor	75 (6.8)
Blood donor, n (%)	
Yes	362 (32.9)
Tried	100 (9.1)
No	639 (58.0)
Organ donor card, n (%)	
Yes	201 (18.3)
Not yet	145 (13.2)
No	755 (13.2)
Hospital stay in the last 6 months, n (%)	341 (32.1)

^†^Some data had missing values, % calculated on available data; missing data were excluded

### Global opinion of patients toward clinical research

One-quarter of respondents had previously participated in at least one clinical study (Table 2). Eighty-five percent of respondents rated the research mission of a university hospital as very important and 84% had a positive or rather positive opinion about clinical research conducted at the hospital. Most respondents (79%) considered that it was justified to ask patients to contribute to producing knowledge that will be useful to other persons. Genetic research was also generally well perceived by patients, with 69% of favourable opinion.

**Table 2:** Respondents’ opinion on clinical research

Variables	Respondents (n = 1125^a^)
Participation in clinical studies during the last hospital stay, n (%)	278 (25.2)
Past participation in clinical studies, n (%)	276 (24.9)
Research is an important mission of a university hospital, n (%)	
Very important	942 (84.5)
Rather important	155 (13.9)
Not important	18 (1.6)
Is it justified to ask patients to contribute to producing knowledge that will be useful to other persons? n (%)	
Definitively justified	879 (79.4)
Partially justified	199 (18.0)
Definitively unjustified	29 (2.6)
What is your opinion about clinical research among patients? n (%)	
Very positive	451 (40.7)
Rather positive	484 (43.7)
Neutral	150 (13.5)
Rather negative	17 (1.5)
Very negative	6 (0.5)
Opinion of genetic research, n (%)	
Favorable	768 (69.3)
Not expressed	305 (27.5)
Unfavorable	36 (3.2)

^a^Some data had missing values, % calculated on available data; missing data were excluded

### Patients’ willingness to participate according to the research design

**Fig. 1:**
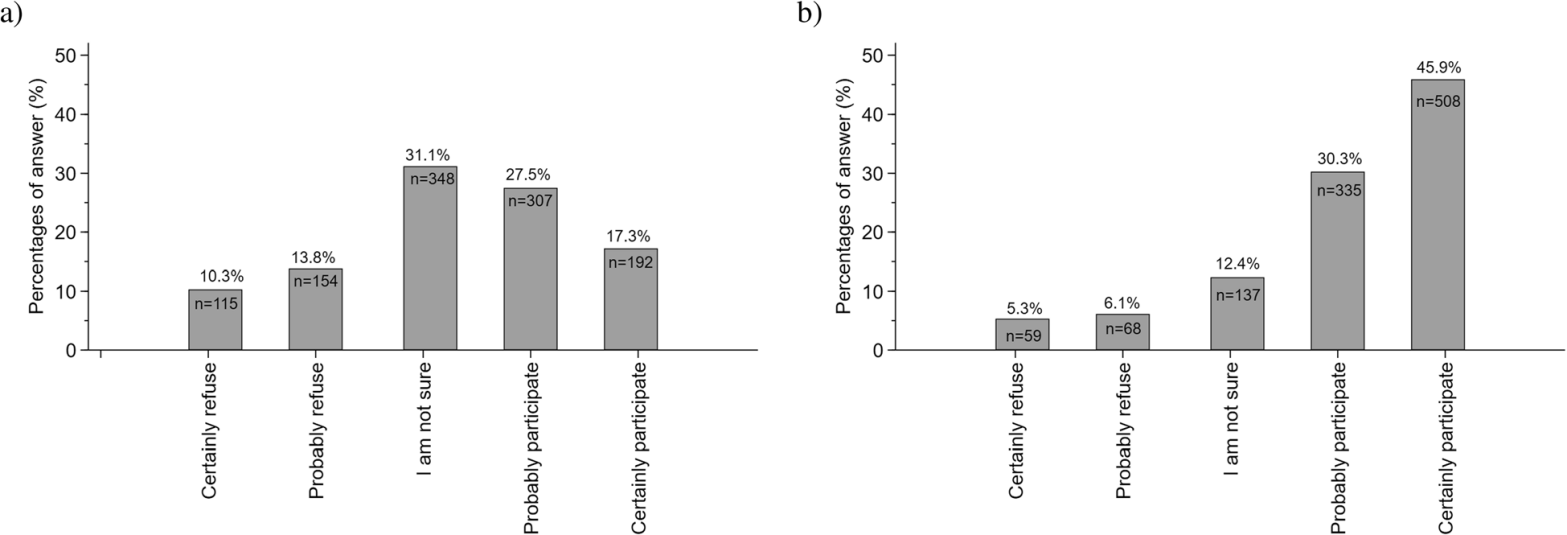
Distribution of the percentages of answers of participants regarding their willingness to be enrolled in a fictive clinical trial assessing the effect of **a** a new respiratory drug and **b** a new diagnostic test

The vignette concerning the drug trial was completed by 1118 (43.0%) participants and the diagnostic study by 1109 (42.7%); 1102 participants (42.4%) completed both. In the drug trial, most respondents answered “*I am not sure*” (31.1%); 44.8% (95% CI 41.9–47.8%) selected that they “*would (probably or certainly) participate*” (Fig. 1a). In the diagnostic study, the percentage of responses increased gradually from 5.3 to 45.9% (Fig. 1b), and the overall willingness to participate was 76.2% (95% CI 73.6–78.7%). Odds of participation in the diagnostic test vignette was 8.1-fold greater (95% CI 6.0–11.1) than in the drug trial vignette (*P* < 0.001). The correlation of answers between the two vignettes was moderate (rho = 0.40).

### Experimental factors associated with willingness to participate

Clinical equipoise and source of funding were significantly associated with the willingness to participate in a clinical trial assessing a new respiratory drug (Table 3). When a medical preference was expressed for the new drug, willingness to participate increased compared to when clinical equipoise was expressed. Public funding also independently increased willingness to participate compared to drug company funding. Random allocation of intervention was not associated with participation. In the diagnostic study, none of the experimental factors was associated with participation.

**Table 3:** Independent associations of six experimental factors with willingness to participate in clinical studies

	Willingness to participate^a^		
Experimental factors assessed in two clinical vignettes	Odds ratio	95% CI	*p-*value
**Vignette 1**			
Belief of greater effectiveness of the new drug (vs. clinical equipoise)	0.77	0.62–0.95	0.013
Random allocation of study drugs (vs. medical decision)	0.99	0.81–1.22	0.949
Public research funding (vs. research financed by a drug company)	1.38	1.12–1.71	0.002
**Vignette 2**			
Rare disease (vs. frequent, heart diseases)	0.95	0.77–1.18	0.660
Genetic analysis of specimens (vs. blood protein analyses)	1.11	0.89–1.38	0.352
Automatic reporting of test results to the participant (vs. reporting only upon request)	1.15	0.92–1.43	0.220

Abbreviation: *CI* Confidence interval

^a^Obtained by ordinal logistic regression model. Willingness to participate was rated on a 5-point Likert scale

### Individual characteristics associated with willingness to participate

When we added the four variables reflecting the patient’s considerations for self and altruistic attitudes in the model, the associations between each experimental factor and participation remained unchanged for the two vignettes (Table 4). In the drug trial, better self-rated health was associated with lower participation (*P* = 0.065). A positive attitude toward research, previous participation in clinical studies and being a blood or organ donor were all associated with increased participation in the trial on a new respiratory drug. In the diagnostic study, a positive opinion toward research, past participation in medical studies and being blood or organ donors were all significantly and independently associated with participation. Excellent or very good self-rated health status was also independently associated with a higher participation compared to good/fair/poor health status.

**Table 4:** Multiple-ordinal logistic regression models per clinical vignette

	Vignette 1^**a**^			Vignette 2^**b**^		
Variables tested	Odds ratio	95% CI	*p-*value	Odds ratio	95% CI	*p-*value
Belief of greater effectiveness of the new drug (vs. clinical equipoise)	0.74	0.60–0.92	0.008	–	–	–
Random allocation of study drugs (vs. medical decision)	1.05	.84–1.30	0.689	–	–	–
Public research funding (vs. research financed by a drug company)	1.29	1.04–1.60	0.022	–	–	–
Rare disease (vs. frequent, heart diseases)	–	–	–	0.90	0.71–1.14	0.387
Genetic analysis of specimens (vs. blood protein analyses)	–	–	–	1.19	0.94–1.50	0.147
Automatic reporting of test results to the participant (vs. only upon request)	–	–	–	1.12	0.89–1.42	0.334
Excellent/very good health status (vs. good/fair/poor)	0.78	0.60–1.02	0.065	1.42	1.07–1.89	0.016
Opinion on research (from “very negative” to “very positive”)	2.38	2.04–2.76	< 0.001	2.71	2.31–3.18	< 0.001
Previous participation in clinical studies (vs. no participation)	1.48	1.14–1.91	0.003	1.45	1.10–1.90	0.008
Blood or organ donor (vs. not)	1.51	1.21–1.88	< 0.001	2.16	1.70–2.74	< 0.001

Abbreviation: *CI* Confidence interval

^a^ Among respondents, 1066 of 1118 (95.3%) had no missing data

^b^ Among respondents, 1056 of 1109 (95.2%) had no missing data

## Discussion

In this experimental study conducted among patients discharged from hospital, willingness to participate in hypothetical studies was lower for a trial assessing the efficacy of a new drug than for an observational study testing the performance of a new diagnostic tool. Participation increased when the new drug was described as likely to be more effective than the old drug (absence of equipoise) or when the study was publicly funded. None of the experimental factors tested in the vignette on the diagnostic study (rare vs. common disease, genetic vs. protein analysis, automatic reporting of results vs. upon request) influenced the patients’ willingness to participate. In both vignettes, patients who reported being blood/organ donors, or who had previously participated in clinical research were more willing to participate. In the diagnostic study, patients who self-reported to be healthier were also more willing to participate.

Most patients had a priori a positive attitude toward clinical research as has been reported previously [8, 16]. The mechanisms underlying willingness to participate integrate the perception of some physical and emotional added values counterbalanced by the expected risks and constraints related to participation in research. All these components are influenced by an individual opinion toward medical care, healthcare providers and the patient’s perception of his/her own health conditions [17]. Nevertheless, in the diagnostic study, we did not see any association between the disease frequency and patients’ willingness to participate, contrary to expectations.

Other factors related to the study itself were associated with participation. Clinical equipoise is the central ethical principle for clinical trials [18]. However, this criterion may be misunderstood by patients, especially if the study investigators are not able to explain its scientific justification. In our study, patients were more willing to participate in the clinical trial when the new drug was presumed to be more effective than when clinical equipoise was presented. Our results conflict with some previous studies. Jenkins et al. reported that the likelihood to participate in a randomised controlled trial was significantly higher among patients with cancer when clinical equipoise was expressed than not [10]. Clinical equipoise justifies the random allocation of treatments but randomisation emerged as a major barrier toward participation in a clinical trial [11] and this method was poorly understood by most patients [19]. Patients preferred physicians to choose their treatment rather than being randomised [10, 20, 21]. In contrast to these studies, we did not see any association between treatment randomisation and willingness to participate, as we previously showed in another survey [22].

The source of research funding could influence the decision to participate in clinical trials. Indeed, we showed that patients were more willing to participate in a publicly-funded compared to an industry-funded trial. Previously, we found a non-significant association between the type of funding and willingness to participate [22]. Similar to the present study, Dias et al. reported that patients were concerned with the maintenance of privacy and safety related to trials and they felt more assured and safe with university-sponsored trials than in privately-funded ones [23]. In another study, patients indicated that they would be more willing to join a clinical trial that was endorsed by national organisations or public institutions [24]. Moreover, unethical behaviour in some drug companies revealed to the general public could explain patient distrust in research funded by pharmaceutical companies [25–27].

We anticipated that willingness to participate in the diagnostic study would be higher when frequent diseases were targeted, as reported elsewhere [28]. However, we did not see such an association. We also expected that patients would be less willing to participate to research in which genetic analyses compared to blood protein analyses were planned, but no such effect was found. In the results of another vignette about the opinion of patients toward biobanking [15], participation was not affected by the type of analyses of the biospecimens. Thus, this lack of evidence for an association does not allow to draw any firm conclusions. We reported here that 69% of respondents had a favourable opinion toward genetic research. Others reported that participation to clinical research is often driven by a doctor-patient trust relationship [16, 21]. Although a high proportion of research subjects wished to receive study results [29], we did not observe any association between automatic reporting of the study results and participation.

The association between altruistic attitudes and participation in clinical trials has been investigated in many studies [4–6, 8, 10–12, 16, 17, 20, 21]. Being a blood/organ donor and having previously participated in clinical trials reflect personal motivations to help others and were associated with willingness to participate in medical research studies [3, 30, 31]. Having a favourable opinion of medical research is also a known predictor for future participation in clinical studies [22]. The association between the patient’s perception of his/her health status and participation in clinical trials has been assessed elsewhere. Patients who perceived themselves to be in good/excellent health were more willing to participate in clinical research than those declaring a good/fair/poor health [22]. By contrast, other authors did not report an association between the respondent’s perceptions of his/her health and participation [3].

The main strength of this study is the use of an experimental design that allowed us to draw valid conclusions on the effect of key study design factors on patients’ willingness to participate in medical research. Moreover, we conducted our survey among former inpatients from a large public teaching hospital who constitute a major pool of research participants in future studies.

Our study has some limitations. First, the participation rate was low, which raises some concerns about the generalizability of the study findings. However, respondents were similar in age and sex compared to overall eligible patients (*n* = 3520). As others have already shown [2], our study participants were mostly women (55.7%), married persons (53.8%) and with a higher level of education (33.1% had attended university or a professional school). Although selection bias is possible in the description of individual characteristics and attitudes toward research, it is less likely for the measures of association between the experimental study factors and reported willingness to participate due to the random attribution of the experimental factors tested. Moreover, as participants were blinded to the manipulation of study factors, it is very unlikely that answers were driven by social desirability. However, it is possible that we would have observed different associations between the experimental factors tested in a different setting and the willingness to participate (effect modification). As a consequence, the generalizability of our study findings is uncertain outside the context of Switzerland. Second, we limited the number of experimental factors tested as only two clinical vignettes were used for this secondary objective of our research project. This decision was motivated by greater study feasibility. Moreover, we acknowledge that other study factors could have been explored, such as the influence of the comparator to new treatment in randomised controlled trials, placebo or standard of care [13], or the expected degree of risks represented by drug toxicities and side-effects of the drugs tested [32].

## Conclusions

In summary, our study confirmed that clinical research is judged by patients as an important mission of a university hospital. Some study attributes were identified as barriers toward participation. The fact that patients were less willing to participate when clinical equipoise was expressed revealed some misunderstanding of a central ethical principle for randomised controlled trials. The lack of association between the random allocation of interventions and participation could be interpreted as a lack of knowledge on another important feature – randomisation - of a clinical trial, but it could also reflect that patients have now accepted this concept. The difficulties to interpret some study findings could be overcome by the use of mixed-method approaches in future research. Priorities for future research should focus on ways to better communicate, particularly on the concepts of clinical equipoise and randomisation.

## Supplementary information

**Additional file 1.** Detailed wording of research vignettes, translated from French.

## Data Availability

The datasets generated and/or analysed during the current study are available in the DRYAD repository (10.5061/dryad.18931zct5).

## Abbreviation

CI: Confidence interval
