# Supplementary material for: Study design factors influencing patients’ willingness to participate in clinical research: a randomised vignette-based study
Source: BMC Med Res Methodol. 2020 Apr 26;20:93. doi: 10.1186/s12874-020-00979-z (PMC7183682; doi:10.1186/s12874-020-00979-z)
Supplement: Supplementary file 1 — Additional file 1. Detailed wording of research vignettes, translated from French. [file 12874_2020_979_MOESM1_ESM.docx]

**Appendix.** Detailed wording of research vignettes, translated from French.

The section starts with the following sentence:

*“Please read the following descriptions of four medical studies. Then, for each study, please indicate whether or not you would agree to participate. These are fictive examples.”*

In this paper, two medical studies were reported; the other two described biobanks.

After each vignette, participants were asked to answer the following question:

“*If you were a patient invited to participate in this study, would you accept or would you refuse to participate?*”

1. *I would certainly accept.*
2. *I would probably accept.*
3. *I am not sure.*
4. *I would probably refuse.*
5. *I would certainly refuse.*

We manipulated three dichotomous factors in each vignettes (a1-a2 / b1-b2 / c1-c2).

Eight versions of each vignette were produced using a factorial design.

**Vignette n°1**

| A new treatment for a lung disease is under evaluation. The treatment aims to improve the patient’s breathing. | |
| --- | --- |
| (a1) Based on previous studies, the researchers believe that the new treatment might be more effective than existing treatments. The goal of the study is to establish whether this is the case. | (a2) Based on previous studies, we do not know what is the best treatment. The study goal is to assess if the new treatment is more or less effective than existing treatments. |
| However, the new treatment can cause digestive problems and sometimes dizziness. | |
| (b1) Patients who accept to participate are allocated by drawing lots, similar to tossing a coin: they have one chance out of two of receiving the new treatment and one chance out of two of receiving the existing treatment. | (b2) The physician in charge of the patient will decide the treatment to be allocated to the patient: either the new treatment or the existing one. |
| The study is financed by | |
| (c1) public research funds. | (c2) a drug company. |

**Vignette n°2**

| A study is conducted at the hospital to evaluate a new laboratory test. This test aims  to better describe the severity of: | |
| --- | --- |
| (a1) some heart diseases, which concern a large percentage of the population. | (a2) the severity of a very rare disease that concerns 1 to 2 persons among 100,000 inhabitants in Switzerland. |
| Patients who participate in the study must have an additional blood test. The goal of this additional blood sample will be: | |
| (b1) to perform genetic analysis testing for specific chromosomic abnormalities. | (b2) to perform blood protein analyses. |
| The study results will be: | |
| (c1) automatically reported to the patients who have accepted to participate. | (c2) only reported upon the patient’s request. |
